# Insights from end-of-career general practitioners on changing working conditions and generational differences: considerations for future strategies

**DOI:** 10.1186/s12875-024-02419-z

**Published:** 2024-05-18

**Authors:** Anne Schrimpf, Elisabeth Scheiwe, Markus Bleckwenn

**Affiliations:** 1 Institute for General Practice, Faculty of Medicine, Leipzig University, Philipp-Rosenthal-Straße 55, 04103 Leipzig, Germany; 2 Institute of General Practice and Family Medicine, University Hospital Bonn, University of Bonn, Venusberg-Campus 1, 53127 Bonn, Germany

**Keywords:** Primary Health Care, Healthcare Reform, Professional Practice, Health Facility Environment, Delivery of Health Care

## Abstract

The landscape of general practice has experienced notable transformations in recent decades, profoundly influencing the working conditions of general practitioners (GPs). This study aimed to examine the most salient changes affecting GPs' daily practices. Through semi-structured qualitative interviews with 15 end-of-career GPs, the study explored how these changes affected work organization, equipment, working hours, work-life balance, job satisfaction, training, patient relationships, and reputation. The interviews revealed that these changes were perceived as barriers, opportunities, or a complex interplay of both for general practice. While the interviewed GPs valued technological advancements and reported positive developments in working conditions, challenges included a gradual reduction in the range of tasks, growing administrative burdens, and less practical training for young physicians. Other changes, such as new doctor-patient dynamics, the transition from single to group practice, and differing professional expectations of the younger generation, were seen as both challenging and strengthening for general practice. By combining these factors and trade-offs observed by end-of-career GPs in our study over the past few decades with general societal changes, we provide ideas for the design of future framework conditions in general practice that might enhance the attractiveness of the profession. These insights offer key considerations that can guide future strategies for general practice and medical education.

**Supplementary Information:**

The online version contains supplementary material available at 10.1186/s12875-024-02419-z.

## Introduction

In just one generation of physicians, the profession of general practice has undergone major changes [1, 2], particularly influencing general practitioners’ (GPs) range of tasks in everyday practice, the dynamic in the doctor-patient relationship, the image of the profession, and their organization of personal and familial commitments [2, 3].

Certain conditions that have emerged are proving beneficial for the profession. Working hours, for instance, have considerably shortened in the last decades and previous long on-call duties for GPs were replaced by introducing emergency medical services (EMS) in the 1970s [4–6]. This change has made the field of general practice more compatible with family life, resulting in an increasing number of women choosing this specialization [7, 8]. Moreover, technical advancements (e.g., computers and practice management systems) have facilitated communication and interaction among colleagues, patients, and health care systems [9].

Other developments, however, might have thrown general practice into a deep crisis. First, GPs’ perceived loss of status among colleagues, students, and patients has been reported as a potential factor discouraging young graduates from entering the field [10–12]. Some attribute this loss of image to the rise of medical specializations and their advancements in diagnostic technologies [13]. The disadvantages are further exacerbated by the disproportionately high volume of administrative tasks relative to comparatively low income of GPs, discouraging young physicians from working in this profession [14–16].

A second crucial point involves the demographic change in Germany and numerous other Western countries that led to a double burden of a greater number of aging or retiring GPs and more older and multimorbid patients requiring resource-intensive treatment and long-term care [17–19]. Since the mid-1990s, GP numbers were declining in Germany [20], precisely at the time when the demand for generalists providing resource-efficient care for the growing number of older patients is starkly increasing [17, 18, 21, 22]. This situation leaves the remaining GPs with an unsustainably high workload, particularly in rural areas [23] and could conceivably aggravate job dissatisfaction and further deter young doctors [24]. Therefore, there is a need for targeted policy actions to invest in health care workers and prioritize their well-being [19].

In conjunction with these developments, the medical field at large is confronted with a sociocultural shift in job expectations: the inclination to subordinate personal needs to the demands of the profession is progressively diminishing among trainees. Young doctors are increasingly vocal about seeking a better work-life-balance, the option of part-time work, and an environment that supports familial commitments [14, 25]. In this context, the ongoing process of feminization of medicine is an element of utmost importance. Over the past several decades, women have increasingly entered the medical workforce. Currently, two-thirds of first-year medical students in Germany are women, and the proportion of female physicians and psychotherapists participating in medical care reached nearly 50% in 2021 [26]. As more young female physicians, and increasingly also their male counterparts, opt for working part-time to balance personal and professional obligations, the future availability of certain medical services might fall short if existing work patterns persist [27–29].

Given these trends, the outlook for working conditions in general practice remains uncertain. This raises the question of how to address the shortage of newly recruited GPs precisely at a time when a stable and efficient primary care system is urgently needed. The aim of this study was to examine which transformations in the profession have had the most profound impact on GPs’ daily practice, which are perceived as beneficial, and which as barriers for work routines. The reflections of end-of-career GPs can provide a link between past and present that is relevant to understanding the evolution of general practice and could guide decisions about future strategies. We conducted in-depth semi-structured interviews with end-of-career GPs who had worked over the last several decades and, thus, were first-hand witnesses to major changes in the profession. With a unique perspective on these changes and their effects on general practice, we particularly investigated perceived changes in various aspects of their careers. We first assessed 1) initial motivation to become a GP, 2) preparedness through training and perceived changes, and 3) daily practice routines and their perceived improvement or deterioration. We also examined changes in relational factors that contributed to or hindered job satisfaction, such as shifts in relationships with 4) patients and 5) colleagues and their impact on professional reputation, and 6) GPs’ work-life balance. In a second step, all reported changes were further synthesized into analytical themes that sought to identify changes that could potentially contribute to a gain in attractiveness of general practice, and those that might deter young doctors in the future and therefore warrant reconsideration.

## Methods

### Participants and setting

The interviews were conducted in Germany between February 2020 and February 2021. Participants were eligible if they were currently working or had worked as a GP and were at least 65 years old. Both purposive and snowball sampling were used to recruit participants [30]. Initially, six GPs were contacted through the researchers’ network. Further, an internet search was conducted to identify local, family-run, and intergenerational rural GP practices. GP practices with websites that listed at least one retired physician in the practice were contacted, resulting in four additional participants. Five participants were recruited using snowball sampling: previous interviewees referred us to their former colleagues and associates. In total, 21 GPs were invited to participate in this study, of whom 15 agreed and six did not respond. A total of two female (13%) and 13 (87%) male GPs participated in the interviews. The mean age of all interviewees was 76 years (range 65–87). The average length of self-employment was 36 years (range 19–45 years). Most GPs worked in single practices (73%) and in rural areas (73%). By the time of the interviews, 47% of the GPs were already retired. Further sample characteristics can be found in Table 1.

**Table 1:** Sociodemographic characteristics of the GP sample

Code	Age	Sex	Practice Established (year)	Type of Practice	Retired (year)	Practice Setting	Federal State of Practice
P01	74	M	1980	Single	Locum	Rural	North-Rhine-Westphalia
P02	80	F	1986	Single	Yes (2005)	Urban	North-Rhine-Westphalia
P03	79	M	1975	Single	Locum	Rural	North-Rhine-Westphalia
P04	81	M	1973	Single	No	Rural	Lower-Saxony
P05	73	M	1978	Group	No	Urban	Baden-Wurttemberg
P06	78	M	1973	Single	Locum	Rural	Lower-Saxony
P07	70	M	1985	Single	No	Rural	North-Rhine-Westphalia
P08	65	M	1991	Single	No	Urban	North-Rhine-Westphalia
P09	70	M	1993	Single	No	Rural	Rhineland-Palatinate
P10	79	M	1972	Single	Yes (2009)	Rural	Rhineland-Palatinate
P11	80	M	1970	Group	Yes (2008)	Rural	Rhineland-Palatinate
P12	87	M	1965	Single	Yes (2000)	Rural	North-Rhine-Westphalia
P13	74	F	1976	Group	Yes (2009)	Rural	Rhineland-Palatinate
P14	84	M	1967	Single	Yes (2008)	Rural	Rhineland-Palatinate
P15	68	M	1983	Group	Yes (2015)	Urban	North-Rhine-Westphalia

*M* male, *F* female, *Locum* working but on a temporary and/or part-time basis

### Data collection and analysis

We utilized a semi-structured interview guideline with five key questions to gain an in-depth understanding of the participants’ former daily practice and perceived changes in relation to today’s conditions. The interview guideline was developed by an interdisciplinary research team (medical scientists and GPs), complemented by a literature review to identify relevant factors [31, 32]. The final guideline can be found in Table 2.

**Table 2:** Final interview guideline

**Key question**	**Target assessment areas of question**	**Questions to receive further information and keep the flow of the conversation (examples)**
How did you get into general practice?	Entry, motivation, training	1. Can you describe in more detail what your medical training was like before you started practicing? 2. Was there a medical background in your family? 3. What was it like for you?
During the first ten years of your practice, what was a typical working day like?	Organization of working hours, everyday family life, leisure time, routines, and deviations	1. How many hours did you work? 2. How did you organize your practice? 3. How did you balance family life and being a doctor? 4. Has anything changed over time? Was it similar to today or what were the differences?
Who have you dealt with on a daily basis in your practice?	Patients, colleagues, employees, and other people	1. How was the collegial environment and cooperation with other employers working in the medical field? 2. Compared to the past, has the relationship with colleagues changed? 3. What kind of staff did you have in your practice? 4. How did the relationship with patients change over time?
What resources did/do you have in your practice?	Diagnosis and range of therapies, priorities, resources, finances, technical equipment	1. What services have you been able to offer your patients in your practice? 2. What changes have you noticed in the range of services during your career? 3. How do you evaluate these changes? 4. What was your financial capacity?
Do you have something else on your mind?	Introduction to the end of the interview, personal message	1. Anything else you would like to say before closing? Any advice you would like to give to young colleagues?

The interviews were conducted by a medical student (ES) under supervision of MB, who is experienced in conducting interviews. The interviewer had no prior relationships with the respondents. Demographic details, including age and sex, years of practice, and retirement status, were recorded prior to the interview. All interviews were audio-recorded with the consent of the participants and lasted between 10 and 60 min, with an average duration of 30 min. Three (20%) interviews were conducted in the respondents’ private homes. In accordance with the COVID-19 safety measures arising during this time, the other twelve (80%) interviews were conducted via phone.

The interview audio files were transcribed verbatim and pseudonymized. To ensure anonymity, recognizable details of the quotes presented in this study were altered (e.g., names of locations). The software MAXQDA 2022 (Verbi GmbH, Berlin, Germany) was used for coding and analysis. The data were analyzed by using a deductive and an inductive approach [33].

We first synthesized the data through line-by-line coding and the development of descriptive themes. For these steps, the questions in the interview guideline were used to develop the main themes. For example, the main categories "Motivation to pursue a career as a GP" and "Medical education" were generated from the introductory question of the interview guideline on career entry. The analysis of the interviews identified in total six main categories, which were generated during the line-by-line coding of the interviews, either indicating new themes or expanding our initial themes of the interview guideline with second-order categories. All categories were iteratively revised, refined, aggregated, or disaggregated during the coding process of subsequent transcripts. In the next step, the derived coding scheme was organized according to contextual coherence and reviewed by two authors. The reliability of the coding was examined by a second rater, who was provided with the first-order categories of the coding scheme and independently re-coded each interview. In the process, a second set of second-order categories was developed and compared with the first set. Disagreements and discrepancies were subsequently discussed with the first rater until an agreement was reached. Results of the inter-rater reliability coding were additionally discussed with other authors. The final coding scheme was then applied recursively to all transcripts. The full descriptive coding tree, including main and subcategories, code definitions, and sample quotations, can be found in Supplementary Material S1.

In a second step, we synthesized the descriptive themes by generating analytical themes [34]. This involved interpreting the primary data within the emerging patterns. All authors of this study agreed on these patterns based on their observations of the descriptive themes.

## Results

### Descriptive themes

#### Motivation to pursue a career as a GP

Eight out of 15 participants stated that their family’s medical background had a significant influence on their decision to become a GP. The wide range of medical tasks available in general practice in the past was another factor that was perceived as highly attractive and motivating for a career in general practice. At the beginning of the participants’ careers (mostly in the late 1970s/early 1980s), GPs’ tasks included more practical activities than today, such as wound care, minor surgeries, knee punctures, and emergency interventions. In addition to the hands-on opportunities offered by the profession, GPs cited trusting relationships with their patients as another driving factor for becoming a GP.

*“As a GP, you could do much more than the internal specialist. I could do bandages; I could do the wound toilet; I could do house calls. I could simply do more practical work as a GP. However, that was in 1980! It was a different time.”* (P1)

*“I want to tell you that we did comprehensive holistic and profound medical care. As it was back then, it is no longer possible today. In fact, you are not allowed to do it anymore. I took care of lacerations; I worked surgically. I had my own little operating room in my practice. [..] That is no longer possible today. I've done intra-articular injections, full-scale leg ulcer therapy, allergy testing, spirometry, and many more.”* (P10)

Other reasons for choosing general practice were the shorter training period compared to specialist training and the possibility of settling down as a GP at a lower cost. At that time, it was possible in Germany to practice as a GP directly after graduating from medical school, whereas today it requires an average of five years to train as a GP after medical school.

*“I worked in surgery for a long time. [..] However, I wanted to be self-employed and considered that in a surgical practice. [..] I would have had to buy much equipment. That’s why I decided to go into general practice instead.”* (P4)

*“I would have had to do another two to three years of training to become a specialist, but not to become a GP. As a GP, I was able to settle down immediately.”* (P5)

#### Changes in medical education and training over time

The interviewees reported continuing progress in training and skills development throughout their careers as GPs. Over time, notable changes in medical education and training were observed. In the past, the interviewees underwent a period called medical assistant before graduating from medical school, a two year practical training in an accredited institution. Surgery and internal medicine were compulsory. After this, they could establish themselves as a GP without further years of specialist training. In particular, the broad practical training was considered to be very instructive and valuable for later establishment as a GP. Over time, practical training has been reduced to one year, which was criticized by the interviewees.

*“Back then, unlike today, there was the medical assistant period. With the state examination you got no license to practice. You first had to complete this assistantship in the main subjects of gynecology, internal medicine, and surgery. Then you got the approbation after about 1 ½ years.”* (P3)

*“Today’s training is actually a disaster. Practice is very different from university. You need completely different things [..] You always have to be in the hospital and with the patient. That's the only way you can learn. You have to see. [..] You can't teach a student [how to recognize acute clinical pictures in practice] if they haven't seen it, e.g., a ruptured stomach. Acute clinical pictures that are really dangerous. You don't forget that [once you've seen it].”* (P13)

#### Changes in everyday practice routines

The interviewees reported a decrease in availability over time. In the past, constant accessibility for their patients was an essential part of their daily routines, consequently leading to a very high workload. Emergency services were scarce in rural areas, prompting patients to call their GP first in case of an emergency. Another aspect was the proximity of the residential building to the practice rooms. It was common for the practice and the living rooms to be situated close to each other, which promoted constant availability as patients tended to contact the GP at home.

*“At that time, the rescue service was not yet organized. They [patients] couldn't call an ambulance. Before people called 112, they first called their GP. [..] I even accompanied the patients to the intensive care unit so that I could verbally inform my colleagues [in the hospital] when I didn't have time to provide them with a brief report.”* (P2)

*“It [practice rooms next door to the living rooms] had advantages regarding going to my workplace. However, people also rang the bell day and night.”* (P10)

The interviewees noted constant changes in diagnostic and therapeutic options. For example, in the past, some medications required closer monitoring due to limited efficacy and potential toxicity. Available medication has improved over time. Further, advancements in technical equipment enabled more accurate diagnosis and therapy. Previously, GPs operated without imaging techniques such as sonography, computed tomography (CT), and magnetic resonance imaging (MRI), making treatment decisions particularly dependent on their own experience.

*“Treatment options were not that good back then. If you had an asthmatic patient back then, the only bronchodilator available was theophylline. We were called at night: ‘Grandpa couldn’t breathe again. Come over quickly.’ Then I drove there with the miracle syringe and injected it slowly, and grandpa got his breath back. [..] Today that doesn't happen anymore, of course, that you get up 2-3 times at night for a patient.”* (P3)

The medical spectrum has also changed over time. Due to the lack of centralized emergency services, some interviewees stated that they engaged in significantly more surgical and trauma-surgical work throughout their professional career than contemporary GPs. Further, most of the older GPs completely replaced the pediatrician in their area and also served as a gynecologist, as home births were common. Some GPs, especially in urban areas, have observed a rise in multimorbid patients over time due to increased life expectancies, which has shifted the spectrum of diseases seen in practice.

*“There was no ambulance back then. For example, if someone fell at school, [..] and had a laceration, we [GPs] would come. They were then stitched up and taken care of. I did bandages and generally did much surgical work. Of course, the entire spectrum of internal medicine came in as well. Heart diseases, lung diseases... I think we did a much broader spectrum than the practices today. I also worked in the pediatric clinic for a while and cared for many children in the practice with check-ups, for example.”* (P4)

Participants elaborated on fundamental changes in general working conditions, such as infrastructure and communication, and their impact on daily routines. The previous lack of telephones impeded communication with the patients and the practice team once the GPs were out on home visits. Further, the poor distribution of transport facilities or ambulances presented challenges to mobility. With the increasing mobility of the patients, the importance of home visits declined, allowing for more practice consultations and, consequently, for better diagnostic possibilities. Additionally, the introduction of computers and digital patient records contributed to improved safety in diagnosis and therapy through fewer misdiagnoses and unwanted drug interactions. However, others expressed a preference for traditional index cards because of their clarity and simplicity. They noted that the introduction of computers made it increasingly difficult to not lose sight of the patient.

*“Making phone calls was so inconvenient in the villages when I made home visits there. There wasn't a telephone in every house. The accessibility was very bad. [..] And in 1976/77 I had a transceiver installed [in my car]. That was very innovative but also expensive. It enabled me to be reached within 20 km of the practice location. That was a great relief.”* (P6)

*“At that time, the population was not as mobile as in the last years of my work. They depended on being cared for at home. It was time-consuming [..]. With time, it changed that more and more could be done in the GP practice. [..] As a result, the doctors around us also made fewer and fewer home visits. At the same time, however, society has become more mobile. The rural society also became wealthier. They then had cars and could come to the practice. [..] Today, we have a completely different level of safety in therapy because you can always call up and control everything. Today you have software where you can enter the symptoms, and the computer tells you whether you can do this or that. Classic misdiagnoses are no longer possible with the technology used today."* (P11)

Other aspects of daily practice involved economic and administrative challenges. Documentation was seen as becoming more time consuming over time. Further, the transformed payment system – from individual billing to a case-based flat rate – prompted changes in the therapy spectrum. Some procedures or services (e.g., home visits) were less profitable and therefore reduced. Several interviewees perceived an intensified economic pressure since their establishment and expressed concern that this could discourage young physicians from settling as a GP.

*“The documentation of our activities used to take up maybe 15% of the time colleagues need today. When I see that every word you exchange with the patient has to be documented, you have to give the young colleagues credit that this is, of course, not beneficial. That takes time. The documentation requirement is outrageous.”* (P10)

*“We had better billing options back then. There were no flat rates, but individual services were billed. I lived through the “golden years” from 1975 to 1985. [..] It got worse in the ‘90s. It always disgusted me that some colleagues cared more and more about the digits when it [the new billing system] became established.”* (P3)

#### Changes in doctor-patient relationships

In the past, GPs described the contact with patients and their families as very close and based on mutual trust. As the technical equipment used to be basic, interpersonal relationships played a pivotal role in diagnosis and therapy. Familiarity with patients’ backgrounds facilitated anamnesis and correct diagnosis. According to most interviewees, diagnosis began with observing and listening. Some interviewees stressed the importance of continuous care and regular GP visits, which facilitated trust and stability. Participating in their patients’ private environment provided valuable information for subsequent diagnoses. The intimacy and attentive care that resulted from these regular visits also had a positive effect on the treatment.

*“I think continuity of treatment is incredibly important. You have to know the patients and the patients have to know the doctor. If you know the grandfather or the father, then maybe you can see where it comes from, I'd say.”* (P4)

*“I know my patients. For me, they are not numbers. If he walks into the room and says I'm really bad, then I can tell you with 90% certainty whether it's serious or not. And that's the difference to a specialist. He doesn't have the comparison. He needs his devices because he doesn't know the patients. That also makes medicine incredibly expensive. If someone comes with knee pain, they are no longer examined at all, but pushed straight to the MRI.” (P7)*

One transformation over time has been GPs’ growing role as mediators. With a rising number of specialists in private practices and hospital specialist departments, GPs assumed the responsibility of educating their patients, as the specialists often had limited time for comprehensive information. However, some GPs worried that patients were progressively relying on these specialists and were losing confidence in general practice.

*“Patients who come from the hospital or are sent to me by a specialist are usually not well informed. I then have much work to explain to the patients what has been done to them and what diseases they have. [..] Ultimately, we are the mediators. Whether it's about delivering bad news or explaining details about the illness to the patient and informing them, we do much educational work, and the patients appreciate that.”* (P7)

*"In the past, when I started, patients got a sick bill, had to see their GP, and they then wrote the referral for the specialist. Today, people race off straight away themselves. Then they complain when they don't get appointments. We used to do most [of the treatment] as GPs. Unfortunately, there is the impression among young people that we GPs can't do anything anymore. We would only write prescriptions. [..] But, as I said, when people have a pimple, they go to the dermatologist. They don't come to the GP anymore."* (P13)

Participants also mentioned changes in patients’ expectations of GPs, particularly that patients were less demanding in the past than they are today. Many interviewees had the impression that GPs currently are under greater pressure to justify themselves and frequently face questioning from their patients. Some were concerned that mistakes could undermine the patients’ trust. This shift was partly attributed to a better education of the patients. The internet enabled them easy access to medical information. This positively affected the cooperation between doctor and patient, but simultaneously introduced new challenges. Doctors had to adapt to patients and their preconceived diagnoses and expectations.

*“I must also say that the demands have changed. In the past, when the doctor came, that was the authority. [..] Nobody got upset about it or went to the BILD [tabloid] newspaper or the police or a lawyer. Today, everyone knows everything about medicine and can read up on it. [..] This makes the doctors very insecure.”* (P13)

*“The bad thing is, [..] that they are all already so pre-educated. They already come to the practice with I don't know how many DD [differential diagnoses], so that you already have such an inner resistance. They don't trust you at all. They want to see a specialist. That is also a fault of the system. GPs are so devalued. They are really only referral writers. [..] The GP no longer has any image at all.”* (P6)

*“Well, I would say the patients are older, more demanding, and better informed. I enjoyed working with informed patients. [..] But I can remember very well that working with patients who had previously googled worked better later. I may have had to correct some things, but overall, the patients were more approachable.”* (P11)

One male interviewee described the importance of female doctors in the doctor-patient relationship. In some cases, patients would prefer to be treated by a female doctor. He believed that female doctors brought a maternal, caring aspect into their daily practice. A female interviewee was critical of the issue of women in general practice, particularly regarding home visits. The situation she might encounter at a patient's home was difficult to anticipate and, as a woman, imposed more risks on her.

*“Our patients got us out of bed at night. [..] That was also a reason for me to stop at 63. I've always said I'm the only old woman roaming the streets at night. [..] We live on the edge of the [location name of a forest], and then in the rain, in the snow, you can't find a license plate or a street sign. You don't know whom you are dealing with. Some drunk people or whatever. [..] I always took my dog ​​with me. I was really scared sometimes.”* (P13)

#### Changes in GPs’ relationship with colleagues and practice structure

In rural areas, GPs described a substantial need for specialist colleagues, requiring them to cover specialist functions. In contrast, relationships with colleagues in urban areas were seen as more competitive, especially with specialists. Communication between practice-based colleagues and those in hospitals was perceived as being more difficult now than in the past. This change was linked to a perceived decrease in personal and transparent communication over time.

*“It [communication] used to be good. [..] They [the specialists] didn't try to take patients away from us. Today it is exactly the opposite. The cardiologist in [location name of a small town] is fully booked for one year in advance because he reorders all the patients. [..] There are so many things being done that are simply not necessary! [..] It didn’t use to be like that. We had our specialists; we knew each other personally. It used to be a different collegial atmosphere. We trusted each other and were not in competition with one another.”* (P6)

*“At least you got a doctor’s letter [from the hospital] relatively quickly [back then]. Or you could just call. Today, the doctors in the hospital don't have any time for such things. [..] Today, the young doctors type everything themselves. It eats up an enormous amount of time and doesn't work.”* (P13)

In the initial stages of the participants’ careers, single practices were particularly common. This setup was associated with a heavier workload, as mentioned by several interviewees. Male GPs often mentioned that their wives were employed in the practice, typically handing administrative tasks. Additionally, other family members often worked in the practice.

*“We old doctors had to work more. My father had a practice with 2500 treatment vouchers [invoiced cases per quarter], he was a so-called cash lion [physician with above-average invoiced treatment vouchers]. If you work together with colleagues in group practice [nowadays], I think you are more economical, more effective, and less burdened.”* (P11)

*“She [his wife] also studied medicine but stopped when we got married, and a child was on the way. She then worked in the practice. I did all the medical parts, and my wife did the organizational part together with the medical assistants. [..] Even when I was away, e.g., for home visits, my wife held the fort in the practice.”* (P4)

Transitioning from single to a group practices or medical care centers was perceived ambivalently. Knowing the patients well and seeing them regularly were perceived to be beneficial for treatment and progression of a disease. For some interviewees, the continuity of care and the personal doctor-patient relationship might be compromised in larger medical care centers. However, the advantages of a medical care center or group practice were also acknowledged, such as lower financial risks, division of labor, direct exchange with colleagues, better working hours, and less personal responsibility.

*“One problem is these medical centers. These are huge practices where the patient sees a new doctor every day. [..] Such things [details of a patient’s medical history] just get lost when five different doctors see the patient in the course of treatment. That was always the strength of GPs [continuous treatment]. [..] With medical centers and employed doctors coming and going, that becomes difficult.”* (P8)

*“You will no longer find many practices where one works alone. Young people will no longer do that, especially since 70% or so are women who also want to start a family. For me, the trend is group practices or medical centers. That will come.”* (P11)

#### Leisure and family time then and now

Reconciling family life, leisure activities, and professional responsibilities emerged as a central theme in the interviews. Some interviewees reported that the practice was a priority and time with the family was limited. Most GPs reported that their wives managed childcare and family life, allowing them to concentrate on their work. Given that many wives also worked in the practices, they frequently employed housekeepers and nannies. One female interviewee specifically emphasized the challenges of balancing work and family as a woman, expressing that she often felt guilty while working.

*“In the past [unlike today], having a practice was the biggest thing. I was really dedicated to my profession 95% of the time. That's where I had my social contacts, too. For me, everything was good with that.”* (P7)

*“We have two girls. They went to the practice after school, and talked and played with the medical assistants. After a while, they went over to the house to eat. In the mornings, we also had a domestic helper who also raised the girls. [..] Everything went through one door; practice and living rooms were directly connected. Even the medical assistants came by briefly, e.g., during lunch, if they needed a signature for a prescription or something like that.”* (P4)

*“After eleven years of parental leave [..] I settled down at the age of 46 [..]. I was only able to do that because the kids distanced themselves a bit, so prepubescent, [..] and the love for my profession came through again, and I thought, man, I have the rooms; I dare to do it. However, I only did this with a very bad conscience because of the children.”* (P2)

Leisure time varied widely between GPs. While some GPs effectively managed a balance with work, others, particularly those in rural areas, found their leisure time substantially limited by professional commitments. Criticism frequently revolved around changing expectations of younger generations who place more value on leisure activities. The interviewees observed that leisure time activities in rural areas were less attractive to today's generation of doctors, which presents a major challenge for the profession, particularly for rural GPs.

*“I don’t know any free time. I was in the tennis club, I was in the choral society, etc., but in the end, I didn't have time for that at all. [..] Work-life balance is an expression that I don't really know. Then the first, second, third child came. We were also busy with that [..]. Otherwise, I have to be honest; there was nothing in terms of free time. Today, yes, you hardly find a life partner who goes along with something like that. One would say: you only live for your job.”* (P7)

*“I always say that when you move to the country with your family as a young doctor, you will soon be alone. The wife runs away, the children run away. The demands have changed.”* (P13)

### Analytical themes

Three patterns emerged from the analysis of the descriptive themes: a) changes that were perceived as positive over the course of the respondents’ careers and potentially beneficial to general practice, b) changes that were perceived as negative over the course of the respondents’ careers and potentially detrimental to general practice, and c) changes that were perceived as ambivalent. Table 3 provides a summary of each analytical pattern, including the themes that were synthesized to form each pattern.

**Table 3:** Summary of analytical themes and their corresponding synthesized descriptive themes

Pattern 1	Pattern 2	Pattern 3
**Perceived positive changes**	**Perceived negative changes**	**Perceived ambivalent changes**
**Improvement in safety in the diagnosis and treatment of patients**	**Decrease in hands-on care**	**Transition from individual to group practice structure**
• Facilitated by technological developments (e.g., CT, MRI, Sonography) • Attributed to the improvement of drugs and the introduction of evidence-based therapies • Attributed to easier communication between patients and physicians, between practices and hospitals (introduction of the internet and mobile phones)	• Attributed to demographic developments (focus on preventive medicine, treatment of chronic diseases, multimorbid patients, psychosomatic disorders, etc.) • Linked to increasing administrative tasks and documentation requirements (clinical paperwork, billing, coding activities) → losing sight of the patients	+ Sharing responsibility in medical and financial aspects, minimizing personal risks + Facilitates exchange with colleagues on a professional level − Reduces the number of practices located decentral, particularly in rural regions − Potential disruption of treatment continuity and in-depth familiarity with patients
**Reduction of workload**	**Reduction of spectrum of care**	**Better informed and assertive patients**
• Facilitated by technological developments (introduction of the internet and mobile phones) • Attributed to structural changes (introduction of an EMS)	• Attributed to increasing specialization (restrictions of tasks) • Attributed to introduction of an EMS • Facilitated by increasing administrative tasks	+ Enhances patient-centered decision-making leading to increased adherence to treatment − Eroding GPs authority (reduced respect for physicians and confidence in medicine, GP only as a service provider)
	**Decrease in practical training hours in medical education**	**Higher demands of young physicians in terms of work-life balance**
	• Attributed to reduced time for practical teaching during everyday clinical practice (staffing shortages, increased patient volume, administrative burdens)	+ Boost job satisfaction and help maintain a stable retention rate in general practice − Potential exacerbation of pressure on medical services, particularly in less appealing regions

## Discussion

The present study investigated changes in the profession of general practice over the past several decades and their impact on GPs’ working conditions by conducting 15 semi-structured interviews with end-of-career GPs. A strong motivator to pursue a career in general practice was the wide range of medical tasks. The GPs interviewed were able to establish themselves after a shorter but more practical university training than today, leading to an early professional and financial independence. In the early stages of their careers, it was common that predominantly male GPs would provide comprehensive, 24/7 medical care for their patients in mainly single practices and, consequently, were considerably relying on their families’ support. The constant availability for their patients resulted in a substantial workload. However, it also contributed to a strong reputation among patients and colleagues in other medical fields. Later, technological advances improved GPs’ working conditions and safety in diagnosis and treatment. Nevertheless, the progressive specialization in medicine, along with increased administrative tasks, gradually reduced the GPs’ range of tasks and spectrum of care, transforming them from main health care providers into administrators and patient managers. A subsequent decline in status of GPs among colleagues and patients resulted in a decrease in job satisfaction. From these descriptive themes, three analytical themes emerged and are discussed below: changes that were perceived as positive, negative, or ambivalent for general practice. From this, we identified areas for improvement. Our findings provide insights into the evolution of the profession of general practice over a generation of doctors, and how this translates given the current GP shortages and the young trainees’ changed expectations.

### Positive changes in general practice from the GPs’ perspective

Some changes were identified as positive for the profession of general practice. Two aspects were particularly noteworthy according to the interviewed GPs: a significant reduction in workload and an improvement in safety in the diagnosis and treatment of patients. Both aspects have been considerably facilitated by various technological developments, e.g., the development of imaging technologies, but also by improvements of drugs and the introduction of evidence-based therapies. In a study from the 1980s, the rise of technology in medicine was also perceived as positive by the majority of retired US GPs [35]. Further, respondents noted that the introduction of the internet and mobile phones has had a positive impact on general practice. It greatly facilitated communication between patients and physicians, but also between practices and hospitals. The perception of most respondents that technological changes – some of which have been very radical – have supported their work throughout their careers could indicate that new and potentially fundamental technological transformations can be implemented in general practice in the future.

Improvements in working conditions were also attributed to structural changes such as the introduction of an EMS. The first EMS in Germany was established at the end of the 1950s [36]. Initially, this service was provided primarily by self-organized GPs who specialized in surgery and focused on the care of accident victims [6]. According to our interviewees, the introduction of an EMS markedly reduced the constant responsibility and availability of GPs, resulting in less frequent 24-h and on-call services and allowing more time for leisure and family. In the process, primary care medicine has become more family-friendly, and more women have chosen to specialize in this field, which is in line with more recent studies [37, 38]. Our results suggest that the changes in working conditions in the profession of general practice might be particularly attractive to young physicians with family ambitions, especially compared to a full-time job in a medical clinic. As a potential strength of general practice, it is in our opinion important to maintain or improve the working conditions of GPs and to promote these benefits to young physicians to address the GP shortage, especially in rural areas.

### Negative changes in general practice from GPs’ perspective

These positive changes came with trade-offs. The interviewees described changes in their working environment that they viewed as negative for general practice and might reduce the attractiveness of the specialty. First, most interviewees reported that the GPs’ spectrum of care is less comprehensive now. During their early professional activity, they considered themselves to be all-purpose practitioners, including emergency medicine and surgical or trauma surgical work. According to the interviewees, in the past, GPs were the primary point of contact for patients in any medical matter. Although the introduction of an EMS significantly reduced night and week-end duty hours [4–6], it gradually – among other reasons – led to a less practical approach of the tasks, contributing to discontent among GPs. In the interviewees’ view, they observed a negative trend from a hands-on and emergency physician to a patient manager and referral writer. For many respondents, a wide and comprehensive range of tasks was a strong motivator for becoming a GP, suggesting that this development could reduce the attractiveness of the profession. This resonates with findings from studies with medical students, who reported seeing general practice as monotonous [11] and not sufficiently challenging [39]. The parallel development of increasing specialization in medicine additionally promoted a feeling of being replaced among the respondents. The specialization in medicine also led to a variety of restrictions of tasks and, consequently, to a reduced range of services starting since the early 1960s in Germany [40]. GPs’ reduced spectrum of practical tasks and increasing specialization can also be placed in the context of overcrowded emergency departments: Increasing numbers of patients with conditions that do not require urgent or complex interventions and could be safely managed by GPs are putting pressure on emergency departments [41, 42]. By integrating GPs more closely into the emergency physician system and reusing more of their resources, the German health system could meet two challenges at once: relieving the pressure on emergency departments and putting GPs back in charge [43].

In addition to changes in the scope of tasks, the GPs in our study noted that other developments, such as increased administrative tasks and documentation requirements, had a negative impact on workload and job satisfaction, which is in line with the literature [15, 24, 44]. A recent study found that a significant proportion of a GP's workload today consists of administrative burdens such as clinical paperwork rather than face-to-face consultations with patients [45]. Considering that physicians’ work dissatisfaction predicted future reductions in work hours [46] and that extensive administrative tasks influenced their intention to leave general practice [15, 47], our findings suggest that reducing administrative work could maintain GPs’ work satisfaction and prevent further attrition.

The interviewees also emphasized that medical education and training used to be more practical than they are today. They described the practical period after their academic training as being longer and more versatile, which enabled them to have a wide knowledge and carry out a broad range of tasks. Indeed, this observation aligns with a review showing that practical training, such as bedside teaching, decreased from 75% in the 1960s to 16% in the 1990s, although it has been shown to significantly increase clinical experience and correct diagnoses [48]. But also further professional training is affected by the reduction in practical hours, which may have an impact on their post-training experience [49]. This might have been exacerbated during the COVID-19 pandemic, which allowed medical students only limited patient contact [50] and might had a negative impact on their clinical skills development. Our results show that although the curriculum of medicine is already both very intensive and extensive, a stronger focus on practical experience might be necessary to improve the hands-on competences of young physicians.

### Ambivalent changes in general practice from GPs’ perspective

The interviewed GPs also reported about changes in general practice that were viewed ambivalently. These included the changes in general practice structures. The single practice used to be the dominant form of practice, which originated in the post-war period when group practices were opposed by the physicians’ association [51]. It was common for the home and practice to be located in the same building or next door to each other. A notable advantage of this model, according to the interviewees, was the in-depth familiarity with patients and their medical history, which fostered an intimate doctor-patient relationship characterized by mutual trust. The development towards group practices and medical care centers has brought both advantages and disadvantages. On the one hand, larger practices offer the possibility of sharing responsibility and facilitating exchanges with colleagues on a professional level, which was also perceived as advantageous in other studies [52] and might be attractive for young medical doctors to consider rural areas [53, 54]. Recent research supports this perception, finding lower work dissatisfaction among GPs working in group practices compared to GPs in single practices [55, 56]. On the other hand, the GPs interviewed expressed concerns that the decline of single practices in favor of group practices would thin out the number of decentrally-located practices, especially in rural areas, exacerbating challenges related to access to health care services, which has also been discussed in the literature [57]. Another trade-off that was criticized by the GPs interviewed, but also elsewhere [52], concerned the potential absence of the patients’ treatment continuity and its negative impact on the doctor-patient relationship in group practices and medical centers due to a large and dynamically changing team of doctors. Indeed, while group practices have been reported to be better equipped and collaborate better with colleagues, patients expressed a greater preference for single practices [58]. Additionally, continuity of care has been shown to affect treatment outcomes, adherence, and the use of preventive measures [59]. This underlines the need for future models of care, probably with a greater shift towards group practices, to take these aspects into account and to introduce doctor-patient routines similar to those in single practices.

Respondents were also ambivalent about changes in the doctor-patient relationship. From their perspective, patients have become both more assertive and better informed, thereby triggering both positive and negative transformations in the relationship. In recent decades, this relationship has evolved from a predominantly paternalistic approach to a patient-centric model in which the patient assumes a more active, autonomous position [60]. While physicians are not inherently opposed to patient-centered care [61], its implementation is perceived as challenging their professional identity and weakening their standing. This could potentially have an impact on patients’ sense of respect for physicians [62]. Indeed, studies have shown an increase in patients’ negative attitudes towards physicians and a decrease in confidence in medicine over time [60, 63, 64]. In general practice, specifically, the better-informed patient might no longer perceive the GP as a counsellor, but rather as a service provider and an intermediary to the specialists [65]. This discrepancy in expectations directly impacts the GP’s role as a medical guide and points to the need to strengthen the role of the GP in the health care system.

Another aspect that emerged from the interviews was the demands of young physicians regarding leisure activities and work-life balance. Initially, the respondents described this development as rather negative. However, between the lines, there were also indications that these developments could benefit young doctors. The demands of the younger generation were described as potentially disadvantageous in the context of recruiting GPs for positions in rural and consequently less attractive areas. The male GPs in our study indicated that they had limited time for leisure or family matters, adhering to the traditional gender-based division of labor. This often meant leaving childcare to their wives or employing nannies – a stark contrast to the expectations of present-day generations [49, 66]. Additionally, the medical workforce has undergone significant transformations over the past several decades, with women now accounting for around two-thirds of those entering the medical profession [26]. While their growing inclination towards part-time employment and reluctance to extend working hours in both male and female young physicians could exert further strain on medical services [27–29], it might simultaneously increase their job satisfaction and contribute to maintaining a stable retention rate in general practice [66].

### Limitations

The limitations of this study should be considered when interpreting its findings and generalizing them to the wider population of end-of-career GPs in West Germany. First, the sample size was relatively small, with only 15 GPs included, potentially limiting the representation of diverse experiences and perspectives. Additionally, the sample composition, comprising two female and 13 male GPs, introduces potential limitations in the applicability of the results to the experiences of female end-of-career GPs, which is why the results cannot be generalized. However, the gender composition observed in our study mirrors the prevailing trend for the participants’ age cohort [67–70], reflecting the low share of women who established themselves as GPs in West Germany between the 1960s and the late 1980s. Specifically, at the beginning of the 1960s, only 14.4% of all established GPs in West Germany were women. This figure rose to 16.9% in the early 1970s and 20.7% in the early 1980s [67, 69, 70]. In line with this, Dettmer et al. [68] reported that the proportion of women working as physicians (independent of specialization) in West Germany was 15.8% at the beginning of the 1960s, 19.2% at the beginning of the 1970s, and 21.6% at the beginning of the 1980s. Additionally, the female share of medical students was reported to be 23% at the beginning of the 1970s and 36% at the beginning of the 1980s [68]. Further, the absence of a comparative group, such as GPs from different age groups or regions, could potentially impede a holistic understanding of the transformations and challenges faced by retirement-age GPs. The limited geographical diversity, primarily rooted in practices in West Germany, could constrain the applicability of the findings to areas in East Germany, where a distinct health care system evolved during the period of the former German Democratic Republic.

Second, the recruitment methodology included purposive and snowball sampling strategies. This approach carries the inherent potential for selection bias and exclusion of certain subgroups of GPs. Additionally, non-response bias from GPs who did not participate could affect the extent to which the findings can be generalized.

Third, relying on retrospective data collection through participant recall might lead to memory bias and inaccuracy. Older individuals have been shown to better recall positive memories than younger individuals [71]. Physicians, in particular, have been found to be nostalgic about aspects of their medical work that were either perceived as at risk or were already disestablished, which has been discussed as a way of preserving their professional identity [72]. Furthermore, self-reported data might be influenced by social desirability or subjective interpretation. Additionally, the cross-sectional nature of this study and the lack of longitudinal data provide only a snapshot of GPs' experiences at one point in time.

### Implications

Our research contributes to a better understanding of how the changes in the profession of general practice in the last decades were perceived by GPs and how they affected their working conditions and job satisfaction. Our results might have general implications that might help to shape future strategies for primary health care and medical education. Considering the mean age of today’s GPs, demographic changes, multimorbidity, and the anticipated decline in treatment capacities on the one hand [17, 18, 22], and the lack of attractiveness of the profession for young doctors on the other hand [15, 39], the future of primary health care is uncertain. By combining these societal changes with the negative and positive factors observed by end-of-career GPs in our study over the past few decades, we highlight potential areas for improvement that could be considered in future strategies (Table 4). We further propose to test the assumptions made in our study in a cohort of young GPs and policymakers, to ensure the applicability of our findings. This could provide insights into the practical challenges and opportunities associated with implementing our recommendations and would allow us to refine our recommendations and ensure that they are grounded in the current realities of contemporary GPs.

**Table 4:** Potential areas for improvement of GPs’ working conditions

(1) **Preserving general practice and professional identity:** The study's findings on the reduction in the comprehensiveness of GP roles over the decades raise concerns about preserving the essence of general practice. The perceived replacement of GPs by specialists and the diminished range of services GPs can provide signal potential challenges for the profession. To address these, the roles of GPs and specialists need to be re-evaluated to promote collaborations that best serve patients' needs while sustaining GPs’ guiding role. We also suggest that expanding the range of tasks in GP practices and reducing administrative and bureaucratic burdens can significantly contribute to less monotonous work and greater job satisfaction
(2) **Strengthening the integration of technological advancements in general practice:** Our findings suggest that most GPs value the ongoing technological progress in their practices. These advancements, particularly those focused on improving patient care quality, streamlining medical procedures, and alleviating administrative burdens on GPs, should be further and rapidly integrated in general practice. However, it is important that GPs participate in the decision-making process and that these implementations are mature and fully functional. Consequently, this could potentially allow GPs more time for patient interaction, which in turn has been shown to increase job satisfaction [15]
(3) **Balancing modern practice structures and care:** Our results suggest that the changes in general practice structures towards group practices and practice centers have professional, economic, and personal benefits for GPs. However, these changes need to be balanced to maintain continuity of care and to prevent the thinning out of GP practices, which would compromise the doctor-patient relationship and make access more difficult, especially in rural areas. We therefore suggest that in less attractive regions with a greater likelihood of more centralized group practices in the future, alternative structures such as telemedicine, shuttles to medical centers, or mobile practices should be implemented. Delegating more tasks to nurse practitioners and physician assistants could be another component of future strategies
(4) **Maintaining the doctor-patient relationship:** The perceived changes in the dynamics of the doctor-patient relationship indicate that the public image of the GPs’ role needs to be adjusted. This could be supported by policies that recognize GPs as central figures in the health care system and allocate resources to strengthen general practice, including better funding for GP practices and infrastructure improvements. In addition, although patient-centered care and shared decision-making are important, the perceived mismatch of expectations between doctors and patients needs further attention. We suggest that enhancing GPs’ communication skills – e.g., during academic and further training – to navigate these changing dynamics could support trust in and continuity of the doctor-patient relationship, while addressing the concerns and needs of both GPs and patients
(5) **Attracting and retaining young physicians:** Given the changing expectations of younger generations, it is imperative to focus on improving working conditions for GPs, particularly in regions facing GP shortages, such as rural areas. Therefore, highlighting part-time work options, autonomy, flexibility, and family-friendly arrangements, on the one hand, and diverse and rewarding aspects of general practice, such as continuity of care and patient relationships, on the other, could strengthen general practice's attractiveness as a career option
(6) **Practical training of students:** The interviewees emphasized the importance of extensive practical training for young physicians, both during their studies and further training. In light of a gradual reduction of practical training in the recent decades [48, 49], strengthening practical experience and interdisciplinary training could better prepare young physicians to meet the diverse needs of patients. We further suggest that integrating a broader range of medical, psychosocial, administrative, and management skills into general practice education and training programs could improve the preparedness of young physicians when getting established in a GP practice

## Conclusion

Our study provides multifaceted insights into the transformations and challenges experienced by retirement-age GPs and their impact on working conditions over the recent decades. While the interviewed GPs reported positive developments in technology, diagnostic and treatment options, as well as in working conditions, challenges included a gradual reduction in the range of tasks and an increase in medical specialization, growing administrative burdens, and less practical training for young physicians. Other changes, such as new doctor-patient dynamics, the transition from single to group practice, as well as differing professional expectations of the younger generation, elicited mixed feelings from the interviewees. The interviews revealed that the landscape of health care has undergone significant transformations and that GPs have made commendable efforts to adjust to these changes. These adaptations have undoubtedly brought benefits in terms of safety, convenience, and accessibility for both doctors and patients. However, our interviews also revealed that these adjustments came with trade-offs, particularly with the loss of the traditional doctor-patient relationship and the professional reputation due to diminished task diversity and increased specialization. Our study provides ideas for the design of future framework conditions in general practice that support the increase and preservation of attractiveness of general practice for young physicians. We suggest that these efforts should focus on preserving GPs’ professional identity by strengthening their role in the health care system. Collectively, these insights provide considerations that can guide the future strategy for general practice and medical education, increasing their attractiveness, and ensuring that general practice remains resilient and able to meet the growing health care needs of the population.

### Supplementary Information

Supplementary Material 1.

## Data Availability

The data that support the findings of this study are available on request from the corresponding author, A.S.
