# Supplementary material for: Insights from end-of-career general practitioners on changing working conditions and generational differences: considerations for future strategies
Source: BMC Prim Care. 2024 May 18;25:171. doi: 10.1186/s12875-024-02419-z (PMC11102275; doi:10.1186/s12875-024-02419-z)
Supplement: Supplementary file 1 — Supplementary Material 1. [file 12875_2024_2419_MOESM1_ESM.docx]

**Supplements**

**Supplementary material S1**. Coding tree, including main and subcategories, code definitions, and sample quotes.

1. **Motivation to pursue a career as a GP**

This category captures the different aspects that led to the decision to become a GP. The category is organized around three subcategories representing the interviewees’ personal reasons and motives.

- 1. Family background and role models: This subcategory highlights the direct influence of a family medical background. This subcategory was assigned when the interviewees stated they chose a career in general practice as a result of their families' ownership of practices.

Example:

*“My father was a GP and so was my brother. It's a tradition in our family. I took over the practice together with my brother. Sometimes the three of us worked together. From a very young age, I was able to witness everything and went on home visits with my father.”* (P13)

- 1. Possibilities of the specialty: This subcategory was assigned when the motivation for choosing general practice was determined by the numerous practical opportunities available at the time. In addition to the practical aspects, this subcategory also included motivations arising from a commitment to patients' holistic well-being and the ability to provide substantial help through personal conversations and attentive listening.

Example:

*“I want to tell you that we did comprehensive holistic and profound medical care. As it was back then, it is no longer possible today. In fact, you are not allowed to do it anymore. I took care of lacerations; I worked surgically. I had my own little operating room in my practice. I treated skin [..]. I was allowed to apply everything I learned. That is no longer possible today. I've done intra-articular injections, full-scale leg ulcer therapy, allergy testing, spirometry, and many more.”* (P10)

- 1. Attractive conditions: This subcategory refers to incentives to choose general practice, such as a comparatively short training period compared to other medical specialties, which offers the opportunity to establish oneself as a GP with relatively little financial investment.

Example:

*“I first worked in surgery for a long time. At that time, we were still doing the medical assistant period, which lasted two years: six months internal medicine, three months gynecology, three months surgery, and I think pediatrics was also mandatory. [..] However, I wanted to be self-employed and had thought that this would only be possible in a surgical practice with the D-doctor system [accident insurance doctor]. I would have had to buy a lot of equipment. And that’s when I decided on general practice.”* (P4)

1. **Perceived changes in medical education and training over time**

This category covers the respondents’ career progression to one’s own practice and self-employment. It also includes the ongoing progress of training and skills development in their daily life as a GP. The category is organized around two subcategories.

- 1. Changes in education and training: This subcategory was assigned when changes in medical education and training throughout the interviewees' careers were mentioned.

Example:

*"Our bosses at the hospital at that time were personalities. They were indeed people you could relate to, and they taught us more of this whole "being a doctor" thing. Nowadays, the young people are put under pressure by the manager to make a profit and that is exactly what the young assistants take over. This empathy for the patients is no longer there. Maybe the [young physicians] do not even have time for that anymore. [..] The [physician] is only seen as a revenue generator. [..] I truly believe that the bosses today no longer convey this image of a doctor. They are all medical professionals, but they are no longer doctors. They're technically proficient in their field and it's dealt with and fine. But it's not the physician doing it comprehensively anymore."* (P6)

- 1. Status and function of further trainings: This subcategory covers the importance for most interviewees of ongoing training for their professional growth, citing various personal benefits. For some, it provided a chance to network, update knowledge, and discuss cases. Others found it valuable for blending work with leisure or spending quality time with family.

Example:

*“There were training courses every Saturday, on Wednesday afternoons, then on Tuesday and Friday evenings. At that time, the pharmaceutical companies subsidized such things. I benefited a lot from that, and it had the advantage that I got to know other colleagues. [..] I often went to the university on Saturdays for very advanced training courses. [..] There I had my problem patients on index cards under my arm. Of course, I would tell the patient that I would like to take the case with me to the training. I then asked the questions in the discussion during the training and got different opinions.”* (P2)

1. **Changes in everyday practice routines**

This category addresses the routines and challenges of the respondents’ professional working day. Besides diagnostic and therapeutic options, the category includes the interviewees’ perceptions of the workload and the transformation of the GPs’ daily routine over time. It is organized around four subcategories.

- 1. Constant availability: This subcategory includes statements about the interviewees’ constant accessibility to patients due to various factors, such as the lack of formal emergency services or the proximity of their homes to their practices.

Example:

*“It [practice rooms next door to the living rooms] had advantages regarding going to my workplace. However, people also rang the bell day and night. But I also had my mobile phone 24 hours a day for 36 years and was always available for my patients.”* (P10)

- 1. Change of the disease spectrum and the diagnostic and therapeutic options: This subcategory includes statements about the continuous development of diagnostic and therapeutic options during the interviewees' careers. Statements about perceived changes in the spectrum of diseases treated were also assigned to this subcategory.

Example:

*"Well, it was quite a wide spectrum at that time. Everything that came up. Like an ambulance. There was no ambulance back then. For example, if someone fell at school, on the sports field or in the ice rink, and had a laceration, we would come. They were then stitched up and treated. I did a lot of bandaging and surgical work in general. Of course, there was also the whole spectrum of internal medicine. Heart disease, lung disease... I think we did a much broader spectrum than the practices do today. I also worked in the pediatric clinic for a while and therefore I took care of a lot of children in the practice with preventive screenings, for example."* (P4)

- 1. Change of infrastructure and the technical equipment: This subcategory was assigned when external factors impacting GPs' daily work, such as mobility, premises, or technology, were discussed.

Example:

*"We made many home visits. That was lifesaving after the war. In many areas, farmers used to pay with butter and cheese. [..] At that time, the population was not yet as mobile as in the last years of my work. They depended on being cared for at home. The good old country doctor came by every three to four weeks and took care of them. In some villages there were also meeting places such as a pub. There the patients all sat together. It was time consuming, but the mileage allowance was well paid. But that was a time when you had to rely on your five senses and what you had in your doctor's bag. A blood glucose meter was added later. That was something special back then. Over time, it became clear that more and more could be done in the general practice. The introduction of ultrasound, ECG [electrocardiogram], and X-ray brought new diagnostic possibilities. As a result, the doctors around us also went to the GP less and less. At the same time, society became more mobile. The society in the countryside also became wealthier. They then had cars and could come to the practice. So that has changed fundamentally in the 42 years I have been working."* (P11)

- 1. Economic challenges: This subcategory covers economic and financial challenges faced by the GPs during their careers and how it impacted their relationship with patients, home visits, and young physicians’ willingness to take financial risks.

Example:

*"My wife is a trained accountant, and she did all the practice billing and bookkeeping. I never took care of it. I disliked doing that. Back then, we had somewhat better billing options. There were no flat rates, but individual services were billed. I experienced the golden years, in quotation marks, from 1975 to 1985. The children all went to university and things were going quite well. In the 1990s it got worse. I was always disgusted that some colleagues cared more and more about the numbers as it became more established. [..] I'm very grateful for everything the way it went. I come from a big family with many children. I didn't bring anything with me. I threw myself in at the deep end. I don't think you could do that today. I had no capital. I took out a loan of 140,000 DM [Deutsche Mark] without having a penny in my pocket. I didn't know how it would turn out. I didn't think about it."* (P3)

1. **Changes in doctor-patient relationships**

This category addresses the relationship between doctor and patient and how it has changed during the respondents' professional life. Important aspects that were mentioned were the change in the position and role of the GP as well as the change in patients’ expectations of GPs. Another aspect was the changing role of women in general practice and its impact on patient care. The category is organized around five subcategories.

- 1. Change of position and role of the GP: This subcategory refers to topics regarding the shifting roles and changing perceptions of GPs, encompassing issues such as young doctors' self-confidence, the weight of responsibilities leading to self-doubt, and the evolving roles of GPs as mediators and educators amidst the growth of specialized fields.

Example:

*“I trained here in an old hospital in [location name]. I have experienced conditions that you as a young doctor can no longer imagine. So, I'll just say that medicine has gotten better. People are more spoiled; they are more demanding. The way my father or I used to work doesn't exist anymore. People used to have basic trust. They didn't have the option of constantly rushing off to some medical specialists. It was also learning-by-doing. Of course, lots of mistakes were made. That's actually not supposed to happen. The error rate must remain very small. But in the past, what self-confidence we had as young doctors. And we knew so little from today's perspective.”* (P13)

- 1. Treatment of patients: This subcategory refers to how patient care in their daily practice changed during GPs’ careers. It covers aspects such as the importance of attentive observation and continuous treatment for trust, the impact of increasing computer use on patient care, and effective communication, especially in sensitive discussions.

Example:

*“The advantage of my practice management was we didn't have computers at that time. Only later because of online billing. But I still had my paper index cards. I always had an eye on the patients. I wanted to see their posture, their facial expressions, how tense or anxious they were. And only when they were out, I quickly wrote everything down."* (P2)

- 1. Changed expectations of the patients: This code refers to changing patient expectations. It encompasses differing views on changes in patient demands, increased scrutiny, and age-related variations in patient expectations, with older patients needing more time and attention, while younger patients seek shorter interactions and referrals.

Example:

*“When I started, the patients got a sick note, had to go to the GP, and he then wrote the referral for the specialist. Today, people rush out themselves immediately. Then they complain when they don't get an appointment. In the past, we did most [of the cases] by ourselves when we were GPs. Unfortunately, it gives young people the impression that we GPs can't do anything anymore. We would only write prescriptions. Actually, it is a very interesting profession because you are confronted with everything. Skin to gynecology, everything. But as I said, when people have a pimple, they go to the dermatologist. They don't come to the GP anymore. And in my opinion, that's also the fault of the health insurance companies. They make it possible for patients to run everywhere with the card. There is no sanction if they see 30 doctors. That is a bit frustrating.”* (P11)

- 1. Improved living conditions of the patients: This code encompasses how patients' changed living conditions affected their relationships with GPs, covering increased mobility, internet-driven patient education, and overall health improvements.

Example:

*“People became more mobile, wealthier, and new opportunities emerged such as patient services: free taxi services. Coming to the doctor's office brought more and more advantages, also in therapy. Think of infusion therapy.”* (P11)

- 1. Women in medicine: This subcategory was assigned when the influence of women in medicine on doctor-patient relationships was discussed. It includes positive perceptions, with patients favoring female doctors for their nurturing approach, and negative concerns expressed by a female interviewee regarding unfamiliar situations during home visits, especially at night.

Example:

*“There was no emergency service. Our patients got us out of bed at night. That was normal. That was also a reason for me to stop at 63. I've always said I'm the only old woman roaming the streets at night. It's not fun at night. We live on the edge of the [location name of a forest], and then in the rain, in the snow, you can't find a license plate or a street sign. You don't know whom you are dealing with. Some drunk people or whatever. [..] I always took my dog ​​with me. I was really scared sometimes.”* (P13)

1. **Changes in GPs’ relationship with colleagues and practice structure**

This category addresses the relationship with colleagues (other GPs and specialists) and employees and included both good collegial cooperation and competitive situations. Further, statements referring to changes in practice structures over time were assigned to this category. The main category can be divided into two subcategories.

- 1. Relationship with other physicians: This subcategory addresses interactions among medical colleagues, including challenges like colleague shortages especially in rural areas, competition and communication issues, and the transformation of communication from personal and direct to impersonal and complex over time.

Example:

*“It [communication] used to be good. We [GPs] sent the patients to them [the specialists] as we thought it was right. They didn't try to take patients away from us. Today it is exactly the opposite. The cardiologist in [location name of a small town] is fully booked for one year in advance because he reorders all the patients and says we have to scan the patients again and see what happened to the left/right ventricle. Nonsense. He recruits his patients and no longer needs referral slips. The same goes for the orthopedist... They think it's great.. And in my opinion, there are so many things being done that are simply not necessary! When I see how often he scans the hearts, such nonsense. That doesn't change in a quarter of a year. And if it does, the patient notices it as soon as he can no longer breathe. That's the problem. It didn’t use to be like that. We had our specialists; we knew each other personally. It used to be a different collegial atmosphere. We trusted each other and were not in competition with one another.”* (P6)

- 1. Practice staff and structure: This subcategory addresses staffing and collegial arrangements in GP practices, including family collaborations, changes in practice models, and the evolving landscape of group practices and medical care centers.

Example:

*“In the meantime, I had the feeling that they wanted to give up the whole professional group of GPs. Large ambulances should be built somehow, and there would then also be a continuous on-call service available, but this whole personal thing would then of course no longer exist. I think that continuity of treatment is incredibly important. You have to know the patients and the patients have to know the doctor. If you know the grandfather or the father, then you might also see where it comes from, I would say.” (P4)*

1. **Leisure and family time then and now**

This category covers all statements related to the organization of GPs’ daily lives outside of practice and work. It is particularly concerned with aspects of reconciling family life, marital life, and personal leisure activities with professional responsibilities and obligations. This category is divided into two subcategories.

- 1. Compatibility of family and work: This subcategory refers to how interviewees managed the balance between their medical careers and family life. It covers the challenges of demanding medical practices, the impact on marriages and families, and the advantages of having medically trained spouses.

Example:

*“We have two girls. They went to the practice after school and talked and played with the medical assistants. After a while, they went over to the house to eat. In the mornings, we also had a domestic helper who also raised the girls. And that actually went quite well. The children really liked that. Everything went through one door; practice and living rooms were directly connected. Even the medical assistants came by briefly, e.g., during lunch, if they needed a signature for a prescription or something like that.”* (P4)

- 1. Leisure activities: This subcategory includes statements about the interviewees' leisure activities, including limited free time, challenges in finding leisure activities in rural areas, and the impact of changing expectations on work ethics and rural living among younger doctors and their partners.

Example:

*“I don’t know any free time. I was in the tennis club, I was in the choral society, etc., but in the end, I didn't have time for that at all. I was only a paying member. In a small town, it was proper to support the associations. I was quite happy to do that, but there were no night services or substitutes for the practice. Or emergency services. You could be reached 24 hours a day. [..] Work-life balance is an expression that I don't really know. Then the first, second, third child came. Yes, ...(laughs). We were also busy with that, if you wanted to see anything of your offspring at all. Otherwise, I have to be honest; there was nothing in terms of free time. Today, yes, you hardly find a life partner who goes along with something like that. One would say: you only live for your job.” (P7)*
